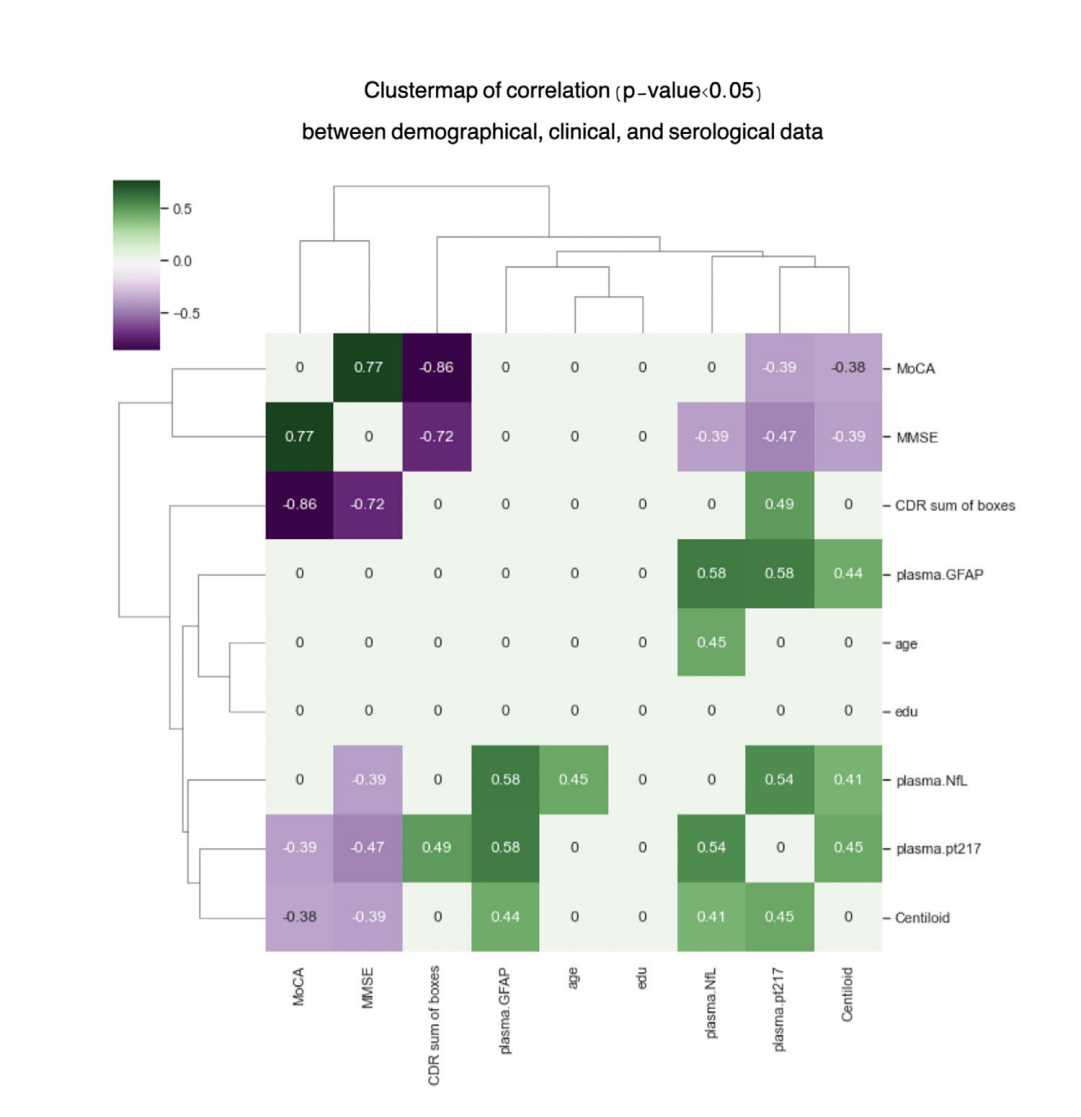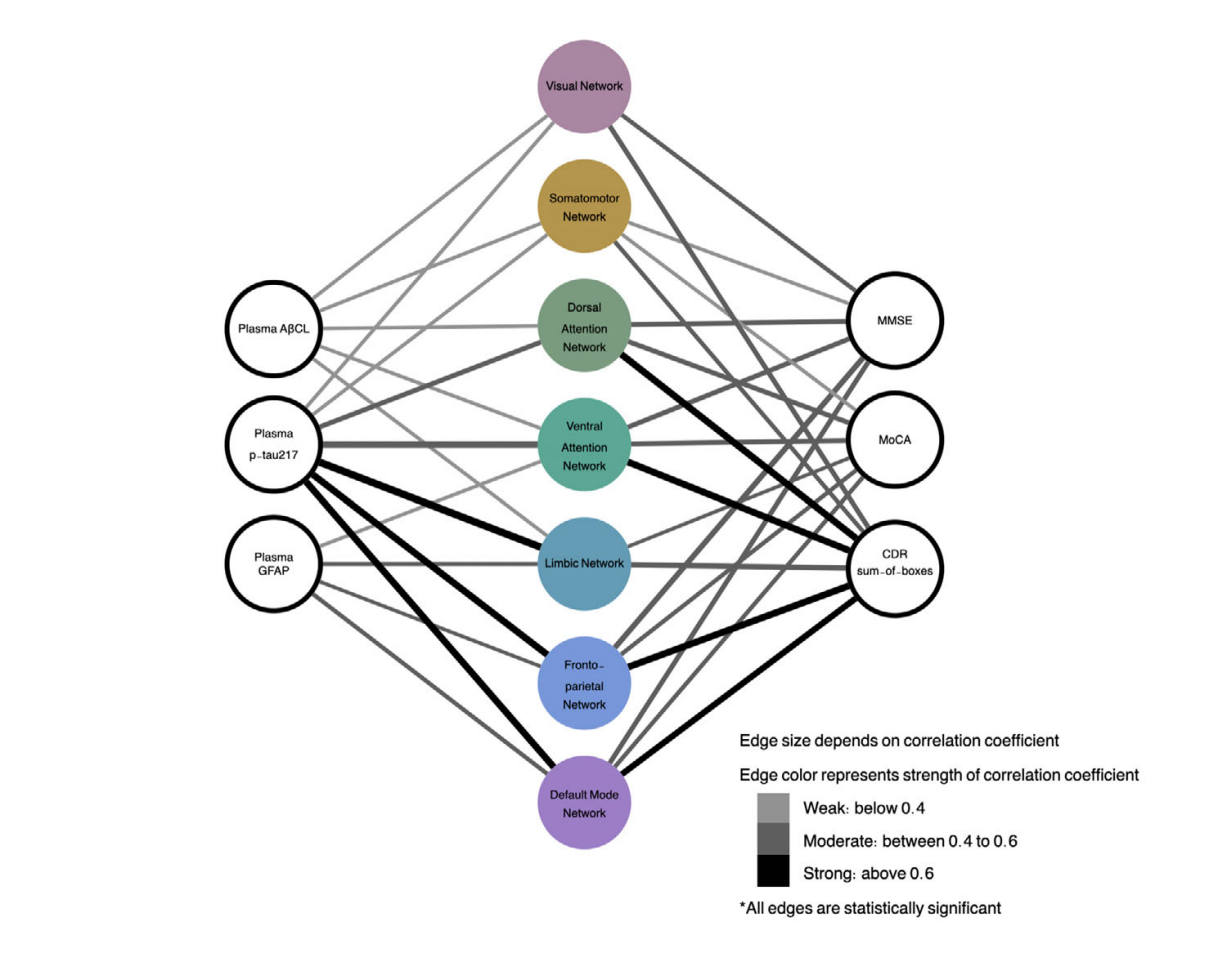# Brain network is greater than sum of its regions: how plasma tau levels associate with tau‐PET limbic network while clinical scores demonstrate strong link with tau‐PET dorsal attentional network

**DOI:** 10.1002/alz.093670

**Published:** 2025-01-09

**Authors:** Setthanan Jarukasemkit, Sekh Thanprasertsuk, Kittithatch Booncharoen, Watayuth Luechaipanit, Adipa Chongsuksantikul, Thirawat Supharatpariyakorn, Yuttachai Likitjaroen, Thiravat Hemachudha, Poosanu Thanapornsangsuth, Chaipat Chunharas

**Affiliations:** ^1^ Cognitive Clinical and Computational Neuroscience (CCCN) Center of Excellence, Chulalongkorn University, Bangkok Thailand; ^2^ Faculty of Medicine, Chulalongkorn University, Bangkok Thailand; ^3^ Neurocognitive Unit, Division of Neurology, Faculty of Medicine, Chulalongkorn University, Bangkok Thailand; ^4^ Thai Red Cross Emerging Infectious Diseases Health Science Centre, King Chulalongkorn Memorial Hospital, Bangkok Thailand; ^5^ Chula Neuroscience Center, King Chulalongkorn Memorial Hospital, Bangkok Thailand; ^6^ Cognitive, Clinical and Computational Neuroscience (CCCN) Center of Excellence, Chulalongkorn University, Bangkok Thailand

## Abstract

**Background:**

Tauopathy is recognized not only as a pathological substrate but also exhibits a robust correlation with the clinical manifestations of dementia, leading to diverse neuropsychiatric manifestation. However, human brain functions as networks rather than modules. The conventional query of 'Where is the lesion (regionally)?’ may inadequately capture the entirety of dementia manifestations. Therefore, direction to focus brain networks, rather than regions, represents nature of human brain and may provide insights beyond blood and regional biomarkers.

**Method:**

In the pilot analysis, amnestic mild cognitive impairment (MCI) or mild dementia (n = 30) were assessed, including demographic, clinical, serological, and neuroimaging data. Clinical metrics comprised Clinical Dementia Rating (CDR), Mini‐Mental State Examination (MMSE), and Montreal Cognitive Assessment (MoCA). Blood‐based biomarkers comprised plasma phosphorylated tau (p‐tau), amyloid‐beta centiloid (AßCL), neurofilament light (NfL), and astrocytic cytoskeleton intermediate filament protein (GFAP). Plasma p‐tau217 and p‐tau181 were measured using Mesoscale Discovery and Quanterix platforms. Position Emission Tomography with a tau tracer (tau‐PET) was used to calculate the standardized uptake value ratio (SUVR) per brain network, according to the Yeo atlas (2011) by Freesurfer. Correlation and statistical differences from network‐based tau‐PET to clinical and serological markers were performed.

**Result:**

Amid of network‐based approach, tau‐PET across all networks correlated significantly with p‐tau, notably with the limbic network displaying the highest correlation (r = 0.76, p<0.01), followed by default mode network (r = 0.73, p<0.01), and frontoparietal network (r = 0.71, p<0.01). Despite this, cognitive scores behaved another way, which moderate‐to‐strong associations were found predominantly in the dorsal attention network, correlations ranged from 0.52 to 0.72 for MoCA, MMSE, and CDR sum of boxes (p<0.01). While dorsal and ventral attention networks demonstrated moderate‐to‐strong correlations with cognitive scores, the limbic network exhibited only weak‐to‐moderate links. Visual and somatomotor networks showed variable correlations with cognition, whereas frontoparietal and default mode networks displayed moderate‐to‐strong links with both serological levels and cognitive scores. Lastly, the network‐based analyzes significantly outperformed the region‐based tau‐PET approach in demonstrating links with cognitive scores.

**Conclusion:**

This evidence highlights the potential of a network‐based approach to demonstrate relationships with patients' cognitive functions, serving as promising biomarkers beyond the constraints of regional‐based biomarkers, and reflecting the true nature of human brain.